# Knowledge, attitudes and practices of health professionals regarding nutrition support in public hospitals of Ehlanzeni district, Mpumalanga

**DOI:** 10.4102/hsag.v31i0.3429

**Published:** 2026-10-05

**Authors:** Phindile P. Khoza, Annette van Onselen, Mashudu Manafe, Reno E. Gordon

**Affiliations:** 1 Department of Human Nutrition and Dietetics, School of Health Care Sciences, Sefako Makgatho Health Sciences University, Pretoria, South Africa; 2 Department of Life and Consumer Sciences, College of Agriculture and Environmental Sciences, University of South Africa, Johannesburg, South Africa

**Keywords:** nutrition support, knowledge, attitudes, practices, multidisciplinary team, hospital nutrition, Mpumalanga, South Africa

## Abstract

**Background:**

Effective nutrition support improves outcomes and quality of life in malnourished patients, yet hospital practices remain inconsistent.

**Aim:**

This study determined and described health professionals’ knowledge, attitudes and practices regarding nutrition support in public hospitals.

**Setting:**

The study was conducted in 13 public hospitals (10 district, 2 regional and 1 provincial) in Ehlanzeni district, Mpumalanga.

**Methods:**

A quantitative, descriptive, cross-sectional study was conducted among 357 randomly selected health professionals using a self-administered questionnaire. Data were analysed with descriptive statistics and binary logistic regression (Stata 13.0).

**Results:**

Only 38% knew that severe malabsorption and undernutrition with poor oral intake indicate parenteral nutrition. Few participants (7.0% strongly agreed, 11.4% agreed) felt that only dietitians are responsible for nutritional care. Although 74% reported that malnutrition was a hospital priority, only 47% screened patients for malnutrition. Education was the most consistent predictor of knowledge, attitudes and practices (*p* < 0.05).

**Conclusion:**

Health professionals had moderate knowledge and positive attitudes, but their practices did not align with this knowledge. Structured training, ongoing mentorship and standardised protocols are recommended to support consistent, evidence-based nutrition care using a multidisciplinary, team-based approach.

**Contribution:**

This study adds to the scarce South African data on hospital-based nutrition support by providing district-level findings from Mpumalanga and highlighting the multidisciplinary nature of nutrition support in public health.

## Introduction

Malnutrition among hospitalised patients is a clinical condition resulting from insufficient intake or utilisation of nutrients, which leads to changes in body composition, diminished physical and cognitive performance and unfavourable clinical outcomes (Cederholm et al. 2019). These patients are particularly at risk due to factors such as disease-driven inflammation, reduced food consumption, impaired nutrient absorption and elevated metabolic requirements (Blaauw 2022; Cederholm et al. 2019; Ramuada et al. 2023). Evidence further indicates that hospitalised individuals frequently receive suboptimal nutritional care, which can aggravate their health status and contribute to the development or progression of malnutrition (Correia, Perman & Waitzberg 2017; Mkhize & Sibanda 2020; Ramuada et al. 2023).

Malnutrition is widespread among hospitalised patients globally, with reported prevalence ranging from 23% to 55% (Marchand et al. 2022). While South African hospital data remain scarce, studies conducted in public hospitals indicate comparably high rates of 48.2% – 54% (Blaauw 2022; Blaauw et al. 2019). Poverty both elevates the risk of malnutrition and exacerbates its consequences (Schneider et al. 2019). These conditions are interconnected in a self-perpetuating cycle: Malnutrition undermines economic productivity and contributes to poverty, while poverty increases susceptibility to malnutrition through food insecurity and restricted access to nutritious foods (Siddiqui et al. 2020).

According to the upper poverty line measure, 1.26 million people in Ehlanzeni District were living in poverty in 2019, representing a 6.21% increase compared with 2009 (Cooperative Governance and Traditional Affairs 2020). This growing poverty burden may contribute to malnutrition among high-risk populations. Nutrition support, delivered by trained health professionals, provides evidence-based nutrients orally, enterally or parenterally to preserve or improve nutritional status (Bally et al. 2016; Lesser & Lesser 2021). It forms a critical component of medical treatment and can substantially affect clinical outcomes in critically ill patients (Hill 2015; Singer et al. 2019).

Higher educational attainment, nutrition-specific training and institutional backing for nutrition care among health professionals are associated with improved patient outcomes, healthier dietary behaviours and a lower risk of nutrition-related chronic diseases (Bjerrum, Tewes & Pedersen 2012; Crowley, Ball & Hiddink 2020). Nonetheless, health professionals frequently find it difficult to apply this knowledge consistently in daily clinical practice, particularly in resource-constrained environments (Stevenson et al. 2018). Insufficient knowledge, suboptimal practices and negative attitudes towards nutrition among health workers are linked to poorer hospital outcomes (Iqbal et al. 2015).

Evidence from African settings highlights ongoing deficiencies in health workers’ nutrition knowledge and practice. In Ghana, Mogre et al. (2017) reported that nurses achieved an average score of only 54.0% on a nutrition knowledge test, demonstrating limited understanding of malnutrition and its management. In Ethiopia, Hadera, Worku and Tuli (2022) found that 67.7% of intensive care nurses had inadequate knowledge of enteral nutrition, and more than half exhibited poor nutrition practices. They identified limited training opportunities, restricted access to protocols and low awareness of guidelines as major obstacles to effective nutrition support (Hadera et al. 2022).

Comparable issues have been found in South Africa. Mooi and Ncama (2020) showed that health professionals in district hospitals and primary healthcare facilities in KwaZulu-Natal had poor knowledge of enteral nutrition therapy, despite generally favourable attitudes and were largely unaware of, and failed to use, national enteral nutrition guidelines, thereby hindering evidence-based nutrition care in the public sector. Likewise, Ramuada et al. (2023) documented inadequate enteral nutrition knowledge among nurses in a South African military hospital, even though they recognised the value of nutrition support.

Collectively, these findings suggest that positive attitudes alone are not enough; effective nutrition care also depends on sufficient training, institutional backing and multidisciplinary teamwork. Optimal nutrition support requires a multidisciplinary model in which all team members participate in nutrition screening, assessment, monitoring and management (Mooi & Ncama 2020). However, most studies in Africa and South Africa predominantly investigate nurses, with limited examination of the broader healthcare team. This has created a knowledge gap regarding the nutrition-related knowledge, attitudes and practices of all professionals involved in nutrition support in South Africa’s public hospitals.

This study addresses this gap by evaluating these aspects among a wider range of health professionals working in public hospitals in the Ehlanzeni district of Mpumalanga, South Africa. The study supports Sustainable Development Goal (SDG) 3 on good health and well-being and contributes to the implementation of South Africa’s National Development Plan (NDP) 2030, which aims to enhance health outcomes and strengthen the health system (United Nations 2015). It is also aligned with the Department of Health’s Integrated Nutrition Programme (INP), which promotes the prevention and management of malnutrition through nutrition screening, assessment, advocacy and support within healthcare services. This study aimed to determine the knowledge, attitudes and practices of health professionals regarding nutrition support in public hospitals in Ehlanzeni district, Mpumalanga province.

## Research methods and design

### Study setting

Mpumalanga is a province located in the north-eastern part of South Africa bordering Mozambique and Eswatini. It comprises three districts, namely Ehlanzeni, Gert Sibande and Nkangala. In 2019, the population of the Ehlanzeni district was 1 856 753, which is 40% of the province’s population and 3.2% of South Africa’s population. The Ehlanzeni district has four subdistricts: Mbombela, Thabachwewu, Nkomazi and Bushbuckridge, and it has 13 public hospitals, with an average of 345 beds. The provincial and/or academic hospital located in the capital city of Mbombela is the biggest in the district and caters for patients who are referred from the district and regional hospitals.

### Design

A quantitative, descriptive, cross-sectional study was conducted to assess nutrition support knowledge, attitudes and practices among health professionals in the Ehlanzeni District, Mpumalanga province, South Africa. This design allowed collection of numerical data at a single point in time, providing a snapshot of the situation and enabling analysis of associations between these outcomes and selected socio-demographic characteristics (Baumeister et al. 2026).

### Population and sampling

The study was conducted across all 13 public hospitals in the Ehlanzeni District, comprising 10 district hospitals, two regional hospitals and one provincial hospital, with a combined bed capacity of 4485 beds (Mpumalanga Legislature 2020). According to human resource records from the Mpumalanga Department of Health, 4997 health professionals were employed in the district as of 01 March 2019. These data were utilised because the study protocol and sampling frame were developed during the same period.

The required sample size was estimated using the Raosoft online sample size calculator, assuming a 95% confidence level, a 5% margin of error and a 50% response distribution, which yielded a minimum required sample of 357 participants. Of the 540 eligible health professionals approached, 430 provided written informed consent and received the self-administered questionnaire. Questionnaires were collected immediately upon completion. In total, 357 questionnaires were fully completed and included in the final analysis. Questionnaires with substantial missing data or not returned because of competing clinical duties were excluded. This corresponded to an overall response rate of 66.1% and a questionnaire completion rate of 83.0%.

### Sampling strategy

Health professionals were recruited through convenience sampling during multidisciplinary meetings. After the study had been explained and informed consent obtained, participants were randomly chosen by drawing numbered lots, and only those who drew the predetermined number were included. A multistage sampling approach involving convenience recruitment is followed by simple random selection. If a selected participant declined to take part, they were replaced using the same random selection procedure to preserve representation from all disciplines. Because participation was voluntary, the study may have been affected by self-selection bias, with individuals who had a particular interest in nutrition being more likely to participate.

### Inclusion and exclusion criteria

The target population was non-dietetic health professionals providing direct patient care in public hospitals in the Ehlanzeni district. Eligible participants were registered speech therapists, speech and audiology practitioners, clinical associates, pharmacists, nurses, dentists and medical officers who were on duty during data collection and provided informed consent. Health professionals were excluded if they worked night shifts, were not present during daytime multidisciplinary meetings when data were collected or were employed on a casual or part-time basis. Students and interns were also excluded.

### Data collection and instrumentation

Data were collected using a self-administered, structured questionnaire adapted from Laur et al. (2016) to assess health professionals’ knowledge, attitudes and practices regarding nutrition support. The instrument was reviewed and modified to align with the study objectives and the South African public hospital context. Changes included adding a socio-demographic section and refining knowledge, attitude and practice items to focus on nutrition support activities such as malnutrition screening, enteral and parenteral nutrition, referral pathways and nutrition-related training. The final questionnaire comprised four sections: One on socio-demographic information and three on knowledge, attitudes and practices related to nutrition support (Online Appendix 1).

A pilot study with 35 eligible health professionals at a hospital in the Ehlanzeni district assessed the instrument’s clarity, relevance, feasibility, completion time and data collection procedures; these participants were excluded from the main sample. Although some reported the questionnaire was lengthy, no items were removed, as all were considered essential.

Data were collected over 6 weeks (April 2022 – May 2022). During multidisciplinary team meetings, the researcher introduced the study and invited eligible health professionals to participate. The questionnaire, administered in English (the primary professional language in South African public hospitals), was completed individually in nurses’ stations, doctors’ rooms and offices to ensure privacy. Average completion time was 25 min – 30 min..

### Validity and reliability

The questionnaire was pre-tested with 35 health professionals to assess feasibility, face validity and reliability. Face validity was established through expert review by nutrition and research methodology specialists and pilot testing among health professionals. Feedback indicated that items were clear, understandable and aligned with the study objectives. Content validity was enhanced by adapting a previously validated questionnaire by Laur et al. (2016) and systematically examining the instrument against the study objectives and literature to ensure comprehensive coverage of knowledge, attitudes and practices in nutrition support. Expert appraisal confirmed the relevance and appropriateness of the items. Internal consistency was assessed with Cronbach’s alpha; the overall questionnaire showed acceptable reliability (α = 0.80) (Norsa’adah, Aminu & Zaidi 2020; Taber 2018). Subscale alphas were 0.20 (knowledge), 0.58 (attitude) and 0.67 (practice). The relatively low coefficients for knowledge and attitude likely reflect the heterogeneous, multidimensional nature of these constructs.

### Data analysis

Data were analysed using Stata version 13 (StataCorp 2013). Descriptive statistics (frequencies and percentages) summarised study variables. Binary logistic regression assessed associations between socio-demographic characteristics (age, education level, occupation) and nutrition support-related knowledge, attitudes and practices. Before analysis, knowledge, attitude and practice variables were recoded according to their response categories. Knowledge items were converted to binary variables based on Yes/No responses. Attitude items on a five-point Likert scale were collapsed into dichotomous variables to enable logistic regression. Practice items (True, False, I do not know) were similarly recoded into binary categories, combining conceptually similar responses. Results are reported as adjusted odds ratios (AORs) with 95% confidence intervals (CIs), and *p*-values < 0.05 were considered statistically significant.

### Ethical considerations

Ethical approval to conduct this study was obtained from the Sefako Makgatho Health Sciences University Research Ethics Committee (No. SMUREC/H/320/2019). Gatekeeper permission was granted by the Mpumalanga Department of Health, the Ehlanzeni District Director, and the chief executive officers of all participating hospitals. The study adhered to the Declaration of Helsinki.

Written informed consent was obtained from all participants before data collection. Participation was voluntary, and participants were informed that refusal or withdrawal would not affect their employment. To reduce coercion, managers did not recruit participants or collect data. Participants could withdraw at any time without giving a reason or incurring penalties.

Confidentiality was protected by assigning unique identification codes and removing personal identifiers from research records. Electronic data were stored on password-protected computers, and hard copies, including consent forms, were kept in a locked cabinet in a restricted-access area. In line with institutional policy, data will be stored securely for 5 years, after which electronic files will be deleted and physical documents shredded.

The study was guided by the principles of autonomy, non-maleficence and justice. Autonomy was upheld through voluntary participation and informed consent. Non-maleficence was promoted by minimising risks and maintaining confidentiality and anonymity. Justice was ensured by equal opportunity to participate and fair, transparent and consistent application of inclusion and exclusion criteria.

## Results

### Socio-demographic characteristics

Table 1 presents the socio-demographic characteristics of the participants.

**TABLE 1:** Socio-demographic characteristics (*N* = 357).

Variables	Frequency (*n*)	Percentage
**Age (years)**		
20–30	118	33.0
31–40	107	30.0
41–50	79	22.0
51–60	46	13.0
Older than 60	7	2.0
**Occupation**		
Nurse	221	62.0
Medical officer	85	24.0
Clinical associate	21	6.0
Speech therapist	4	1.0
Speech and audiologist	4	1.0
Pharmacist	14	4.0
Dentist	8	2.0
**Work experience (years)**		
≤ 4	125	35.0
5–10	100	28.0
11–20	78	22.0
21–30	36	10.0
≥ 31	18	5.0
**Place of employment**		
Provincial hospital	93	26.0
Regional hospital	71	20.0
District hospital	193	54.0
**Current station**		
Outpatient department	75	21.0
Wards	211	59.0
Emergency unit	46	13.0
Wellness or HIV clinic	25	7.0
**Duration at current station**		
0–11 months	139	39.0
1–5 years	132	37.0
6 years and more	86	24.0
**Highest level of education**		
Grade 12	31	9.0
Higher certificate	61	17.0
National diploma	107	30.0
BTech or degree	117	33.0
Honours degree	30	8.0
Master’s degree	8	2.0
Doctorate degree	3	1.0
**Institution of higher learning attended**		
Government college	133	37.0
Private college	60	17.0
University	153	43.0
University of technology	11	3.0
**Place studied**		
Within South Africa	318	89.0
Outside South Africa	20	6.0
Both	19	5.0
**Type of nutrition support seen the most**		
Oral	297	83.0
Enteral	23	6.0
Parenteral	21	6.0
None	16	4.0

The study comprised of 357 participants, the largest proportion (33.0%) of which were 20–30 years old, and most were nurses (62.0%). A total of 125 (35.0%) of the participants had four or less years of working experience, 54.0% were employed at a district hospital, 59.0% worked in wards and 83.0% reported the oral route as the most seen type of nutrition support (Table 1).

### Nutrition support knowledge

Table 2 and Table 3 present the participants’ nutrition support knowledge, with correct responses shown in italics.

**TABLE 2:** Nutrition support knowledge (***N*** = 357).

Question	Possible options to answer question	Responses	
		Frequency (*n*)	Percentage
Which of the following is a poor indicator for nutrition status	*Body mass index*	*203*	*57.0*
	Mid upper arm circumference	154	43.0
Indication of enteral nutrition includes and are not limited to the following	Non or semi-functional GIT	53	15.0
	*If a patient does not finish 30% of RDA orally*	*43*	*12.0*
	Dysphagia	86	24.0
	Non- or semi-functional GIT, if a patient does not finish 30% of RDA orally and dysphagia	150	42.0
	Non or semi functional GIT and dysphagia	25	7.0
Indication of parenteral nutrition includes and are not limited to the following	Severe malabsorption	64	18.0
	Accessible GIT	14	4.0
	Severe undernutrition with poor oral intake	61	17.0
	Severe malabsorption, accessible GIT and severe undernutrition with poor oral intake	82	23.0
	*Severe malabsorption and severe undernutrition with poor oral intake*	*136*	*38.0*

Note: Present the participants’ nutrition support knowledge, with correct responses shown in italics.

GIT, gastrointestinal tract; RDA, recommended dietary allowance.

**TABLE 3:** Nutrition support knowledge (*N* = 357).

Statement	True		False		I do not know	
	Frequency (*n*)	Percentage	Frequency (*n*)	Percentage	Frequency (*n*)	Percentage
Nutritional status influences the outcome (morbidity & mortality) in the critically ill	314	88.0	29	8.0	14	4.0
It is mandatory to monitor glycaemic control in patients receiving early enteral or parenteral nutrition	278	78.0	36	10.0	43	12.0
All critically ill patients require individualised treatment by a dietitian	300	84.0	39	11.0	18	5.0
Overfeeding is not a problem with patients on intensive care as the energy requirement are significantly high	43	12.0	286	80.0	28	8.0
Patient’s position when feeding is very important	175	49.0	114	32.0	68	-
Importance of positioning during feeding	328	92.0	11	3.0	18	-
Complications of enteral and parenteral nutrition can be fatal	300	84.0	36	10.0	21	-

Just over half of the participants (57.0%) understood that body mass index is a poor indicator of nutritional status. The majority (84.0%) were aware that complications arising from enteral and parenteral nutrition can be fatal. Only a small proportion (12.0%) recognised that one indication for enteral nutrition is when a patient consumes less than 30% of the RDA orally. In addition, most participants (88.0%) knew that nutritional status affects outcomes in critically ill patients. Fewer than half (49.0%) were aware that a patient’s position is important during feeding. This finding appears inconsistent with the fact that most participants (92.0%) reported that positioning is important during feeding. The overall composite score for nutrition support knowledge was 66.0% (Table 2 and Table 3).

### Nutrition support attitudes

Participants’ attitudes towards nutrition support are summarized in Table 4.

**TABLE 4:** Nutrition support attitudes (*N* = 357).

Statement	Strongly agree		Agree		Neutral		Disagree		Strongly disagree	
	Frequency (*n*)	Percentage	Frequency (*n*)	Percentage	Frequency (*n*)	Percentage	Frequency (*n*)	Percentage	Frequency (*n*)	Percentage
Enteral nutrition supplements induce diarrhoea	64	18.0	113	31.5	91	25.5	14	4.0	75	21.0
Nutrition is not important to every patient’s recovery in hospital	22	6.0	42	12.0	18	5.0	137	38.4	138	38.6
Monitoring food intake is a good way to determine a patient’s nutritional status	145	40.6	153	43.0	22	6.1	9	2.5	28	7.8
Nutritional care of patients is only the role of a dietitian	25	7.0	41	11.4	40	11.0	88	25.0	163	45.6
Measurement of patients’ weight is not necessary on discharge	14	4.0	31	8.6	43	12.0	89	25.0	180	50.4
Survival of critically ill patients improves when evidence-based guidelines for nutrition are followed	143	40.1	136	38.2	47	13.2	9	2.5	21	6.0
Interruptions (including by giving treatment) negatively affect patients’ food intake	56	15.7	130	36.5	63	17.7	15	4.2	92	25.8
All staff involved in patient care can provide hands-on assistance with eating when necessary	117	32.8	151	42.3	57	16.0	3	0.8	29	8.1
If a patient can eat orally, then they don’t need nutrition support	19	5.0	64	18.0	24	7.0	69	19.3	181	50.7
Only a doctor can refer a patient for nutrition support	34	9.5	85	23.8	33	9.0	74	20.7	131	37.0

Only a small proportion (7.0% and 11.4%) strongly agreed or agreed that providing nutritional care to patients is solely the responsibility of a dietitian. The largest share of respondents (32.8% and 42.3%) indicated that all staff involved in patient care offer direct assistance with eating when needed. Similarly, the highest proportion (40.6% and 43.0%) strongly agreed or agreed that tracking food intake is an effective method for assessing a patient’s nutritional status. Fewer than one in four participants (9.5% and 23.4%) strongly agreed or agreed that only a doctor is permitted to refer a patient for nutrition support (Table 4).

### Nutrition support practices

When asked how often they referred patients to dietitians, just over half of the participants (51.0%, *n* = 182) stated that they rarely did so, 38.7% (*n* = 138) reported always referring and 10.4% (*n* = 37) indicated that they never referred patients. In terms of screening for malnutrition, fewer than half of the participants (46.8%, *n* = 167) reported that they routinely screened patients, 31.1% (*n* = 111) said they sometimes conducted screening and 22.1% (*n* = 79) stated that they did not screen patients for malnutrition.

Table 5 outlines the participants’ nutrition support practices of the health professionals.

**TABLE 5:** Nutrition support practices.

Statement	True		False		I do not know	
	Frequency (*n*)	Percentage	Frequency (*n*)	Percentage	Frequency (*n*)	Percentage
Every patient’s nutritional status is assessed at the outpatient department	164	46.0	146	41.0	47	13.0
Malnutrition is a priority in this hospital	265	74.0	46	13.0	46	13.0
I play a role in promoting a patients’ dietary intake	303	85.0	47	13.0	7	2.0
If permitted, I encourage a patient’s family to bring food from home for the patient	182	51.0	154	43.0	21	6.0
I check the patient during their mealtime to see how well they are eating	229	64.0	107	30.0	21	6.0
Patients’ nutrition status is assessed before discharging them from the hospital	243	68.0	71	20.0	43	12.0
Assessment of patient nutrition status during the hospital stay is a regular procedure	250	70.0	64	18.0	43	12.0
Health professionals involved in nutrition screening and assessment undergo training on nutrition support	164	46.0	125	35.0	68	19.0

The majority (74.0% and 85.0%) stated that malnutrition is considered a priority in their hospital and that they are involved in encouraging patients’ dietary intake. Nonetheless, only slightly more than half (51.0%) indicated that, when allowed, they advise patients’ families to bring home-prepared food for the patient. Most participants (68.0% and 70.0%) reported that patients’ nutritional status is evaluated prior to discharge and is routinely monitored during their hospital stay. In contrast, fewer than half (46.0%) reported that health professionals responsible for nutrition screening and assessment receive training in nutrition support (Table 5).

### Logistic regression for occupation, education and nutrition support knowledge

Table 6 presents the results of a binary logistic regression analysis which examined whether occupation and education, predicted nutrition support knowledge.

**TABLE 6:** Logistic regression examining factors associated with nutrition support knowledge.

Statements	Odds ratio	s.e.	*p*-value	95% CI
1. Nutritional status has an effect on outcome (morbidity and mortality) in the critically ill				
Age	1.187	0.200	0.308	0.853–1.652
Occupation	1.099	1.043	0.319	0.912–1.323
Education	0.763	0.083	0.013*	0.616–0.945
2. It is mandatory to monitor glycaemic control in patients receiving early enteral or parenteral nutrition				
Age	1.037	0.200	0.848	0.710–1.515
Occupation	1.232	0.133	0.054	0.996–1.524
Education	0.616	0.084	0.000*	0.471–0.806
3. All critically ill patients require individualised treatment by a dietitian				
Age	0.878	0.192	0.556	0.571–1.351
Occupation	0.643	0.136	0.037*	0.425–0.974
Education	0.998	0.144	0.991	0.540–1.246
4. Over feeding is not a problem with patients on intensive care as the energy requirements are significantly increased				
Age	0.938	0.221	0.789	0.591–1.490
Occupation	1.023	0.183	0.897	0.720–1.454
Education	1.487	0.246	0.017*	1.075–2.058
5. Complications of both enteral and parenteral nutrition can be life-threatening				
Age	1.151	0.238	0.496	0.767–1.726
Occupation	0.946	0.132	0.695	0.719–1.245
Education	0.733	0.104	0.030*	0.554–0.970

CI, confidence interval; s.e., standard error;

* *p*-value statistically significant.

Occupation made a statistically significant contribution for statement number three (*p* = 0.037, OR = 0.99 and 95% CI [0.42, 0.97]). Education was the predictor for most of the statements (one, four and five), (*p* = 0.013, OR = 0.76, 95% CI [0.61, 0.94]), (*p* < 0.001, OR = 0.61, CI [0.47, 0.80]), (*p* = 0.017, OR = 1.48, 95% CI [1.07, 2.05]) and (*p* = 0.030, OR = 0.73, 95% CI [0.55, 0.97]) (Table 6).

### Logistic regression for age, occupation and education and participants’ attitude

The results of a binary logistic regression on whether age, occupation and education, predicted attitude of the participants is presented in Table 7.

**TABLE 7:** Logistic regression examining factors associated with attitude.

Statements	Odds ratio	s.e.	*p*-value	95% CI
1. Enteral nutrition or supplements induce diarrhoea				
Age	0.996	0.160	0.985	0.726–1.367
Occupation	1.267	0.133	0.024*	1.031–1.557
Education	1.322	0.143	0.010*	1.68–1.637
2. Nutrition is not important to every patient’s recovery in hospital				
Age	0.873	0.177	0.505	0.587–1.299
Occupation	0.916	0.124	0.519	0.702–1.195
Education	1.681	0.245	0.000*	1.262–2.239
3. Nutritional care of a patient is only the role of the dietitian				
Age	0.610	0.126	0.018*	0.405–0.917
Occupation	1.477	0.377	0.126	0.895–2.437
Education	1.838	0.285	0.000*	1.356–2.491
4. Measurement of a patient’s weight is not necessary on discharge.				
Age	1.185	0.288	0.486	0.734–1.911
Occupation	1.106	0.185	0.547	0.796–1.535
Education	1.403	0.232	0.040*	1.014–1.942
5. Survival of critically ill patients improves when an evidence-based guidelines for nutrition are followed				
Age	0.645	0.133	0.035*	0.429–0.969
Occupation	0.909	0.126	0.493	0.692–1.193
Education	0.603	0.085	0.000*	0.456–0.796
6. Interruptions either by giving treatment during meals negatively affect patient food intake				
Age	0.836	0.133	0.263	0.611–1.143
Occupation	1.208	0.116	0.050*	0.999–1.460
Education	1.117	0.117	0.290	0.909–1.373
7. All staff involved in patient care can provide hands-on assistance to eat when necessary				
Age	0.773	0.152	0.194	0.525–1.139
Occupation	1.460	0.146	0.000*	1.199–1.778
Education	0.845	0.111	0.203	0.653–1.094
8. If a patient can eat orally then they don’t need nutrition support				
Age	1.009	0.191	0.962	0.695–1.464
Occupation	1.099	0.168	0.535	0.814–1.485
Education	1.789	0.249	0.000*	1.361–2.352
9. Only a doctor can refer a patient for nutrition support				
Age	0.731	0.141	0.105	0.500–1.067
Occupation	3.539	1.227	0.000*	1.794–6.983
Education	1.825	0.261	0.000*	1.378–2.416

CI, confidence interval; s.e., standard error;

* *p*-value statistically significant.

Age proved to be a predictor for statements (three and five), (*p* = 0.018, OR = 0.61, 95% CI [0.40, 0.91]) and (*p* = 0.035, OR = 0.64, 95% CI [0.42, 0.96]). Occupation made a statistically significant contribution for statements (one, six, seven and nine), (*p* = 0.024, OR = 1.26, 95% CI [1.03, 1.55]), (*p* = 0.050, OR = 1.20, 95% CI [0.99, 1.46]), (*p* < 0.001, OR = 1.46, 95% CI [1.19, 1.77]) and (*p* < 0.001, OR = 3.53, 95% CI [1.79, 6.98]). Education was the predictor for statements (one, two, three, four, five, eight and nine), (*p* = 0.010, OR = 1.32, 95% CI [1.06, 1.63]), (*p* < 0.001, OR = 1.68, 95% CI [1.26, 2.23]), (*p* < 0.001, OR = 1.83, 95% CI [1.35, 2.49]), (*p* = 0.040, OR = 1.40, 95% CI [1.01, 1.94]), (*p* < 0.001, OR = 0.60, 95% CI [0.45, 0.79]), (*p* < 0.001, OR = 1.78, 95% CI [1.36, 2.35]), (*p* < 0.001, OR = 1.82, 95% CI [1.37, 2.41]) (Table 7).

### Logistic regression for age, occupation and education and participants’ practices

The findings of a binary logistic regression, which was conducted to examine whether age, occupation and education predicted the participants’ practices is presented in Table 8.

**TABLE 8:** Logistic regression examining factors associated with practices.

Statements	Odds ratio	s.e.	*p*-value	95% CI
1. Do you screen all patients for malnutrition on admission?				
Age	0.659	0.118	0.021*	0.463–0.938
Occupation	0.949	0.096	0.612	0.778–1.158
Education	0.983	0.114	0.886	0.783–1.234
2. Every patient’s nutritional status is assessed at the outpatient department (OPD).				
Age	0.842	0.209	0.491	0.517–1.371
Occupation	1.112	0.124	0.341	0.893–1.385
Education	1.355	0.212	0.053	0.996–1.843
3. Malnutrition is a priority in this hospital.				
Age	0.784	0.207	0.358	0.467–1.316
Occupation	1.251	0.139	0.044*	1.005–1.557
Education	1.153	0.193	0.394	0.830–1.601
4. I check a patient during their mealtime to see how well they are eating.				
Age	0.924	0.325	0.825	0.464–1.842
Occupation	1.310	0.169	0.037*	1.016–1.688
Education	1.807	0.397	0.007*	1.174–2.780
5. Patients’ nutrition status is assessed before discharging them from the hospital.				
Age	0.963	0.265	0.893	0.562–1.652
Occupation	1.720	0.192	0.000*	1.381–2.142
Education	1.136	0.201	0.470	0.802–1.609
6. Assessment of patient nutrition status during the hospital stay is a regular procedure.				
Age	0.446	0.141	0.011*	0.240–0.829
Occupation	1.523	0.171	0.000*	1.221–1.900
Education	1.139	0.217	0.493	0.784–1.656

CI, confidence interval; s.e., standard error;

* *p*-value statistically significant.

Age was a predictor for statements (one and six), (*p* = 0.021, OR = 0.65, 95% CI [0.46, 0.93]) and (*p* = 0.011, OR = 0.44, 95% CI [0.24, 0.82]). Occupation was a predictor for statements (three, four and five), (*p* = 0.044, OR = 1.25, 95% CI [1.00, 1.55]), (*p* = 0.037, OR = 1.31, 95% CI [1.01, 1.68]) and (*p* < 0.001, OR = 1.72, 95% CI [1.38, 2.14]). Education was a predictor for statement number four (*p* = 0.007, OR = 1.80, 95% CI [1.17, 2.78]) (Table 8).

Based on this study’s findings, health professionals demonstrated a moderate level of nutrition support knowledge, with an overall composite score of 66%. Most participants understood the importance of nutritional status in critically ill patients, recognised the role of evidence-based nutrition practices and acknowledged the need to promote patients’ dietary intake. They generally expressed positive attitudes towards nutrition support, and many indicated that nutritional status is routinely evaluated during hospitalisation and prior to discharge. Nonetheless, several key gaps emerged, including insufficient understanding of the indications for enteral and parenteral nutrition, low rates of malnutrition screening and referrals to dietitians and inadequate nutrition support training among health professionals.

## Discussion

This study examined the knowledge, attitudes and practices of health professionals regarding nutrition support in public hospitals in the Ehlanzeni district of Mpumalanga. In contrast to earlier research that focused mainly on nurses, medical officers or pharmacists (Mahmoodpoor et al. 2021; Ramuada et al. 2023; Salih 2016; Shakhshir & Alkaiyat 2023), this study encompassed a wider spectrum of health professionals, including pharmacists, speech therapists, audiologists and dentists. This multidisciplinary focus underscores the inherently collaborative nature of nutrition support services.

Most participants had been at their current station for 11 months or less, primarily because nursing staff are rotated annually within the district. Although job rotation has been linked to higher job satisfaction and engagement among nurses in Greece (Platis et al. 2021), frequent moves may reduce the chance to build in-depth skills in specialised areas such as nutrition support. In the context of the South African public healthcare system, where resources and staffing are under considerable strain (Manyisa & Van Aswegen 2017), the effect of rotation on clinical competence may not mirror that observed in high-income countries.

With respect to nutrition support knowledge, over half of the participants correctly recognized BMI as a less accurate indicator of nutritional status than mid-upper arm circumference (MUAC). This contrasts with findings from Iraq and Malaysia, where only 14.7% and 15.7% of participants, respectively, identified the appropriate indicator (Karim et al. 2015; Salih 2016). This result is promising, as MUAC is a simple, low-cost and dependable screening measure that supports early detection of patients in need of nutritional support, particularly in settings with limited resources (Degarege, Degarege & Animut 2015; Miele et al. 2021; Van Tonder et al. 2019).

In this study, when respondents were questioned about various indications for enteral nutrition, only a small number correctly identified that patients who consume less than 30% of their RDA meet the criteria. This contrasts with findings from a study conducted at a military hospital in Pretoria, where most participants (71%) recognised the correct indication for enteral nutrition (Ramuada et al. 2023). This result is worrisome, as insufficient knowledge regarding indications for enteral nutrition may lead to delays in initiating nutritional support, thereby potentially elevating the risk of malnutrition and negative clinical outcomes.

In addition, fewer than half of the participants in this study were able to correctly identify all indications for enteral nutrition, and only 38% correctly recognised the indications for parenteral nutrition. Comparable gaps in knowledge of nutritional support have been documented in other African contexts. Mooi and Ncama (2020) reported that healthcare professionals in KwaZulu-Natal had poor overall understanding of enteral nutritional therapy, especially in relation to clinical guidelines, monitoring procedures and the management of complications. Similarly, Hadera et al. (2022) found that nurses working in intensive care units in Ethiopia possessed inadequate knowledge of enteral nutrition. Collectively, these results indicate that limited knowledge of nutrition support remains a widespread issue across healthcare settings in Africa.

Most participants in this study indicated that monitoring glycaemic control in patients receiving enteral or parenteral nutrition is essential. This is promising, as the significance of glycaemic management in such patients has been previously emphasised (Polavarapu, Pachigolla & Drincic 2022). Although there is no consensus in the literature on the optimal method for monitoring blood glucose in critically ill patients, studies consistently report that both hypoglycaemia and hyperglycaemia are linked to increased mortality risk (Fong, Au & Ng 2022). While these observations align with evidence largely derived from high-income healthcare systems, the South African setting has distinct challenges. Public hospitals often function under considerable resource limitations, including heavy patient loads and staff shortages (Chitha et al. 2024). As a result, achieving and maintaining adequate glycaemic control may be more difficult than in better-resourced environments, even when healthcare professionals are aware of its importance.

A conflicting result emerged in relation to patient positioning during feeding. Although the majority of participants (92%) reported that positioning is important during feeding, fewer than half (49%) correctly recognised that a patients position during feeding is very important. This inconsistency may indicate that participants have a general awareness of positioning as a component of routine patient care but lack more detailed clinical understanding of its specific role in ensuring safe and effective nutrition support. It may also point to a gap between theoretical awareness and practical clinical knowledge, underscoring the need for more targeted nutrition support training for health professionals.

The overall knowledge score of 66% reflects a moderate level of nutrition support knowledge among participants, similar to that reported for health workers in Qatar, Kuwait and the United States (Vrkatić et al. 2022). While this points to an adequate basic understanding, the gaps observed in particular aspects of nutrition support may nonetheless influence clinical decision-making and patient outcomes.

In terms of nutrition support attitudes, most participants in this study disagreed that only a doctor can refer a patient for nutrition support. In countries such as Austria and the Netherlands, only medical officers are allowed to refer patients to dietitians (Eglseer & Bauer 2020), but in United Kingdom, the United States and South Africa, any licenced healthcare provider can refer a patient to a dietitian (Ramuada et al. 2023).

Most participants in this study agreed that monitoring food intake is a good way to determine a patient’s nutritional status, suggesting that there is a strong awareness among health professionals of the importance of routine dietary monitoring in identifying patients at risk of malnutrition. This aligns with the findings of Laur et al. (2016), which reported that Canadian health professionals recognised that monitoring food intake is a key aspect of determining a patient’s nutritional status.

Furthermore, half of the participants strongly disagreed that measurement of a patient’s weight is not necessary on discharge, which is encouraging given that weight loss is a common and clinically significant problem among hospitalised patients (Marchand et al. 2022). These findings are contrary to that of Shakhshir and Alkaiyat (2023), in which only 19% of participants believed that weight must be measured on patients’ discharge.

Most participants in this study indicated that the survival prospects of critically ill patients improve when evidence-based nutrition guidelines are applied. This aligns with earlier research that highlighted the importance of using evidence-based guidelines for nutritional support in hospitalised patients (Karim et al. 2015; McClave et al. 2016). A notable concern in this study, however, is that fewer than half of the participants reported having received training in nutrition support. Although this proportion is higher than in some earlier studies for instance, research conducted in Pretoria found that only 25% of nurses had training in nutrition support (Ramuada et al. 2023), and it remains suboptimal. Similarly, in Iran, only 8.2% of nurses were reported to have such training (Mahmoodpoor et al. 2021). Ongoing training is essential to strengthen the competence and practices of health professionals, reduce the burden of malnutrition and ultimately improve health outcomes.

Fewer than half of the participants in this study conducted malnutrition screening for their patients, which is concerning because such screening is crucial in both primary and secondary healthcare settings. It facilitates the identification of risk factors and, when nutrition-related issues are found, allows for prompt intervention, thereby helping to reduce the impact of malnutrition-related health complications (Mikkelsen, Geisler & Holst 2022).

In this study, about half of the participants seldom referred patients to a dietitian. This is contrary to the finding regarding the knowledge of the participants as most acknowledged the importance of individualised treatment by a dietitian (Table 4). This finding highlights a knowledge-practice gap, whereby health professionals recognise the value of dietetic input but do not consistently translate this knowledge into referral practices. Referral ensures that patients get the best care possible, minimising treatment gaps and improving the patients’ overall experience (Seyed-Nezhad, Ahmadi & Akbari-Sari 2021).

In examining participants’ attitudes and behaviours in this study, an inconsistency emerged: While most respondents indicated that they assess patients’ nutritional status before discharge, fewer than half reported actually conducting malnutrition screening. This is concerning as unintentional weight loss is a key indicator of malnutrition, and not screening patients may result in missed opportunities for timely intervention (Marchand et al. 2022; Mikkelsen et al. 2022). In addition, the South African National Guidelines on Nutrition Counselling, Support, and Treatment for Malnourished Individuals stipulate that all patients should undergo nutritional status assessment, as this is essential, among other reasons, for generating information to guide the development of an appropriate nutrition care plan (Department of Health 2015).

The gap between what participants state they do and what they actually do can be understood using the Capacity, Opportunity, Motivation and Behaviour (COM-B) model, which posits that behaviour is shaped by capability, opportunity and motivation (West & Michie 2021). Although participants showed moderate knowledge of nutrition support, the observed shortcomings in practice point to barriers that extend beyond knowledge alone. Issues such as heavy workloads, staff shortages, organisational culture and inadequate institutional backing may constrain opportunities to follow recommended procedures. This explanation aligns with emerging evidence from South Africa and Nigeria, where organisational policies and technological infrastructure have been identified as important determinants of behaviour change among health professionals (Anusi & Dlamini 2025).

From the logistic regression analysis (Table 6, Table 7 and Table 8), it is evident that education was the most consistent predictor of knowledge, attitudes and practices of nutrition support among health professionals. Participants with higher levels of education were significantly more likely to provide correct responses to knowledge and attitude items and report positive nutrition support practices. This finding is consistent with Mogre et al. (2017), who similarly reported that prior nutrition training and exposure to clinical guidelines were associated with higher knowledge scores.

Furthermore, the association between education and nutrition support knowledge, attitudes and practices may be explained by higher educational attainment being linked to more extensive clinical training, greater exposure to evidence-based guidelines and stronger critical assessment skills, which together improve the integration of nutrition support into routine practice. Advanced education may improve the knowledge, skills, attitudes and outcomes of clinical practice of healthcare professionals (Aryee et al. 2024; Hill et al. 2024; Howard, Diug & Ilic 2022).

### Strengths and limitations

A key strength of this study was the inclusion of all public hospitals in the district, which improved institutional representation across district, regional and provincial levels of care. Nevertheless, even with this extensive facility coverage, the findings should be interpreted cautiously. The study was confined to a single district in Mpumalanga and focused exclusively on public hospitals, which may restrict the applicability of the results to private healthcare facilities or to other provinces in South Africa. The use of a self-administered questionnaire may have introduced self-reporting and social desirability biases. In addition, the questionnaire was administered in English, which may have affected how some participants interpreted particular questions. The cross-sectional design of the study also limits the ability to draw causal inferences. Finally, although random sampling was applied among those who consented to participate, volunteer bias may still have occurred.

## Conclusion

This study adds to limited South African evidence on health professionals’ knowledge, attitudes and practices regarding nutrition support by providing detailed district-level data from public hospitals in Mpumalanga. Including several professional categories highlights the multidisciplinary nature of nutrition support in the public sector.

Health professionals in Ehlanzeni district public hospitals showed moderate knowledge and generally positive attitudes. They recognised nutrition support as important for improving outcomes, avoiding overfeeding and managing glycaemic control in critically ill patients. However, their clinical practice did not fully reflect this knowledge, especially in malnutrition screening and referrals to dietitians. Although most were aware of evidence-based guidelines and supported a multidisciplinary approach, fewer than half routinely screened for malnutrition or made appropriate referrals, revealing a gap between theory and practice.

This study recommends structured training, ongoing mentorship and standardised protocols to align knowledge with practice and promote consistent, evidence-based nutrition support and better outcomes. Health professionals should use a team-based approach, drawing on diverse expertise to guide each patient’s care. Future research could examine how hospital administrators influence nutrition support.
